# Molecular Interactions Between Components of the Circadian Clock and the Immune System

**DOI:** 10.1016/j.jmb.2019.12.044

**Published:** 2020-05-29

**Authors:** Sophia Hergenhan, Stephan Holtkamp, Christoph Scheiermann

**Affiliations:** 1 Walter-Brendel-Centre of Experimental Medicine, University Hospital, Ludwig-Maximilians-University Munich, BioMedical Centre, Planegg-Martinsried, Munich, Germany; 2 University of Geneva, Centre Médical Universitaire (CMU), Department of Pathology and Immunology, Geneva, Switzerland

**Keywords:** circadian, rhythm, inflammation, immune system

## Abstract

The immune system is under control of the circadian clock. Many of the circadian rhythms observed in the immune system originate in direct interactions between components of the circadian clock and components of the immune system. The main means of circadian control over the immune system is by direct control of circadian clock proteins acting as transcription factors driving the expression or repression of immune genes. A second circadian control of immunity lies in the acetylation or methylation of histones to regulate gene transcription or inflammatory proteins. Furthermore, circadian clock proteins can engage in direct physical interactions with components of key inflammatory pathways such as members of the NFκB protein family. This regulation is transcription independent and allows the immune system to also reciprocally exert control over circadian clock function. Thus, the molecular interactions between the circadian clock and the immune system are manifold. We highlight and discuss here the recent findings with respect to the molecular mechanisms that control time-of-day-dependent immunity. This review provides a structured overview focusing on the key circadian clock proteins and discusses their reciprocal interactions with the immune system.

## Highlights

• The immune system is under control of the circadian clock.
• Circadian clock proteins act as transcription factors controlling genes of the immune system.
• Circadian clock proteins engage in direct physical interactions with inflammatory proteins.
• Immune factors also reciprocally exert control over circadian clock function.

## Graphical abstract

**Figure:**
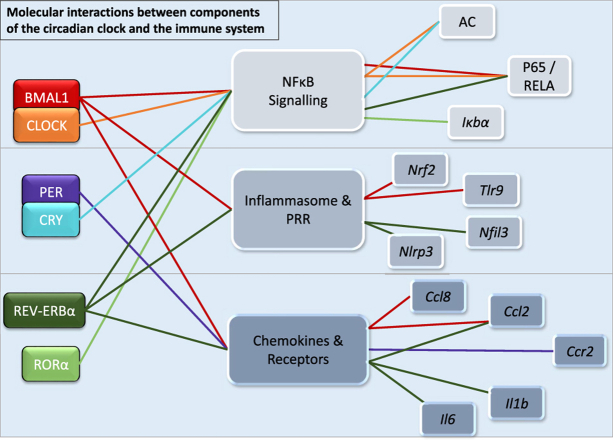

## Introduction

Virtually every organism continuously faces daily and seasonal environmental changes. Organisms anticipate and respond to these changes by tuning their behavior, metabolism, and also their immune system accordingly. Daily, circadian rhythms (from *circa diem*, “about a day”) in physiology provide a means of environmental anticipation and impose oscillations in a myriad of biochemical pathways [1]. Within different organs, approximately 10%–20% of the mammalian transcriptome are under direct circadian control, signifying that these genes experience a peak in expression once every 24 h [[2], [3], [4]]. However, oscillations are surprisingly tissue-specific with very little overlap between organs. This indicates that a much higher percentage of genes can be expressed in a circadian manner in some part of the body. This temporal programming is concerted by intrinsic biological clocks, time-partitioning mechanisms within cells, which are present in most organisms [5].

In mammals, the circadian clock is made up of one master clock and many peripheral clocks. The master clock consists of neurons residing in the suprachiasmatic nucleus (SCN) of the brain. It is situated above the optic chiasm and receives environmental information of light and darkness via the eyes and the associated retinohypothalamic tract [6]. Peripheral clocks are found within cells outside the SCN, including all leukocyte subsets. The master as well as the peripheral clocks are cell-autonomous, i.e., they oscillate continuously. However, peripheral clocks require the master clock for their synchronization as otherwise oscillations between cells become desynchronized over time and flatten out in multicellular organisms [6]. The master clock entrains peripheral clocks via systemic factors. It itself is synchronized by the environmental lighting conditions and thus ensures that the body is in phase with the environment. Light is the main *Zeitgeber* for the master clock (from the German “time giver”) and sets the behavioral activity and rest phase of the organism and the times of feeding. Food is an important external environmental *Zeitgeber* for peripheral clocks that synchronize rhythmicity in different tissues such as the liver [7,8].

Both the master and peripheral clocks share essentially the same molecular architecture [9]. It consists of several interlocking transcription-translation feedback loops. The core transcription feedback loop comprises two basic-helix-loop-helix PER-ARNT-SIM (PAS) domain activators, BMAL1 (brain and muscle ARNT-like 1; encoded by *Arntl*) and CLOCK (circadian locomotor output cycles kaput) [10]. BMAL1 and CLOCK form a heterodimer that binds to Enhancer (E)-box sites located within promoter regions and induces the expression of other clock genes and clock-controlled genes. Among these are the PER (period) and CRY (cryptochrome) clock proteins, which, being analogous to BMAL1:CLOCK, heterodimerize in the cytoplasm (PER:CRY) and translocate to the nucleus, where they inhibit BMAL1:CLOCK-mediated transcription. A new cycle commences when this PER:CRY repressor complex decreases, owing to reduced levels of BMAL1:CLOCK [11]. A second feedback loop consists of the nuclear receptors: RAR-related orphan receptor (RORα,β,γ) and REV-ERB (α,β; encoded by *Nr1d1* and *Nr1d2*) [[12], [13], [14]]. By binding to receptor-related orphan receptor response elements (ROREs) in the promoter region of *Bmal1*, RORs activate and REV-ERBs repress the expression of *Bmal1* in a competitive fashion to ensure a finetuning in expression [13,15]. A third loop includes the expression of the transcriptional activator albumin D-box binding protein (DBP), which is directly regulated through BMAL1 binding to its E-box, and the repressor nuclear factor interleukin 3 (NFIL3; also known as E4BP4). NFIL3 is an important factor for the development of innate lymphoid cells (ILCs) and Th17 cells in the gut [16,17] and is transcriptionally regulated via RORE elements. Both factors together act as transcription factors by binding to D-box elements in genes such as *PER*.

In addition to this tripartite system, control of the clock is achieved via different posttranslational modifications such as—among others—phosphorylation, mediated by kinases (e.g., the casein kinases (CK) CKIα, CKIβδ, and CKIε) and phosphatases (PP1 and PP5), along with histone modifications (CLOCK itself is a histone acetyltransferase (HAT) [18]) and epigenetic manipulations [19,20]. Using internal *Zeitgebers* such as glucocorticoids and temperature, as well as other cues, output that is generated by the hypothalamus-pituitary-adrenal (HPA) axis and the autonomic nervous system (ANS), the SCN orchestrates peripheral clocks in immune cells and organs to ensure a temporal coordination of the physiology within the whole multicellular organism. Furthermore, within the blood circulation reactive oxygen species (ROS) have recently been discovered to play a major role in the species-specific synchronization of leukocytes [21]. Thus, a highly complex network entrains cells of the immune system to the rhythms of the environment.

**Fig. 1:**
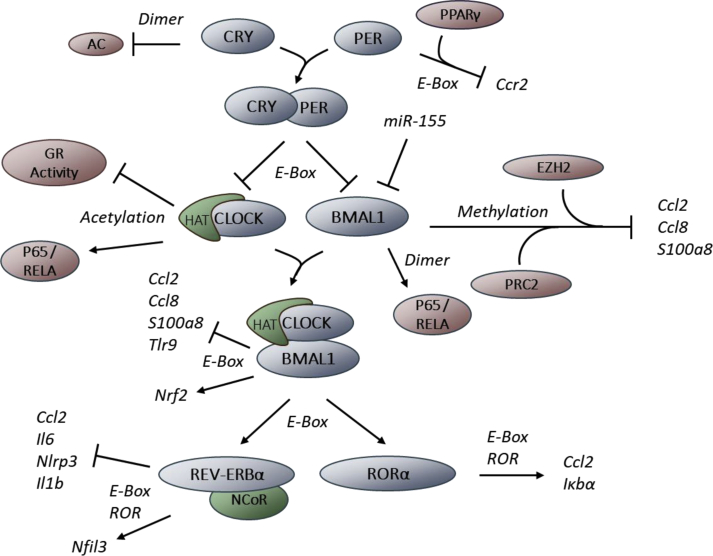
**Molecular connections between components of circadian clocks and the immune system**. BMAL1, in a heterodimer with CLOCK, represses the expression of CCL2, CCL8, S100a8, and TLR9 by binding to E-box motifs. BMAL1 also recruits the Polycomb repressor complex 2 (PRC2) to the promoter of these genes. The histone methyltransferase EZH2 (a member of PRC2) induces the trimethylation of histone H3 at lysine 27 within the *Ccl2*, *Ccl8,* and *S100a8* promoter region leading to reduced transcription. BMAL1 is also able to dimerize with RelB, thus blocking a subunit of the proinflammatory transcription factor NFκB. On the contrary, BMAL1 positively controls the antiinflammatory protein NRF2. The histone acetyl transferase CLOCK acetylates the RelA subunit (NFκB) and glucocorticoid receptors, thereby regulating their DNA binding capacity. The transcription of BMAL1 and CLOCK is under direct control of the repressors CRY and PER. Additionally, translation of BMAL1 is inhibited by miR-155. PER binds together with PPARγ to an E-box in the *Ccr2* promoter region, downregulating its transcription. CRY dimerizes with the adenylyl cyclase (AC) to inhibit its function. BMAL1 upregulates the transcription of the two metabolic genes *Nr1d1* (REV-ERBα) and RORα via E-Box motifs. RORα upregulates the transcription of *IκBα*, the major transcriptional inhibitor of the NFκB signaling pathway, as well as *Ccl2*. REV-ERBα binds histone deacetylase 3 (HDAC3) and the nuclear hormone corepressor (NCoR) to inhibit the transcription of *Ccl2*, *Il-6*, *Nlrp3,* and *Il1b* while at the same time upregulating the transcription of *Nfil3* via E-box and ROR elements.

The clock machinery was found to be expressed and oscillating in all leukocyte subsets investigated thus far, including innate and adaptive immune cells, such as monocytes, natural killer (NK) cells, neutrophils, eosinophils, macrophages, mast cells, dendritic cells (DCs), CD4^+^ and CD8^+^ T cells, as well as B cells [[22], [23], [24], [25], [26], [27], [28], [29], [30], [31], [32], [33]]. Furthermore, many recent publications have shown that immune cell function and dynamics are strongly influenced by the circadian clock [22,27,31,[33], [34], [35], [36], [37]]. In this review we focus on the molecular interactions between the major components of the clock (BMAL1, CLOCK, PERs, CRYs, REV-ERBs, and RORs) and the immune system (Fig. 1). We will furthermore discuss how the immune system can affect the circadian clock in a reciprocal manner. A functional, rhythmic clock in immune cells confers an immunoprotective, healthy state across the whole organism [31]. Genetic disruption of the clock can lead to malfunctioning immune responses [27,33,35] and inflammation [22,28,31,38]. Thus, understanding the molecular mechanisms that link the clock with immune functions is of essence for the proper understanding of the immune system and the exploration of new therapeutic avenues for treating immune pathologies.

## BMAL1

BMAL1 is a central component of the mammalian clock. Most studies have focused on BMAL1, since inactivation of this gene is a convenient way of abolishing clock function by acting only on a single gene, while for the other components of the circadian clock described below multiple genes need to be targeted. Thus, care should be taken in distinguishing BMAL1-specific effects from downstream effects where general clock function is important. Global *Bmal1*^*−/−*^ mice exhibit impaired locomotor activity under rhythmic light-dark cycles. Keeping these mice under constant darkness leads to further loss of circadian behavior and molecular circadian rhythms [39]. Moreover, *Bmal1*^*−/−*^ mice show accelerated aging and mortality and develop progressive arthropathy [40]. With respect to the immune system, *Bmal1*^*−/−*^ mice exhibit impaired B cell development with reduced B cell numbers in blood and spleen and reduced mature B220^hi^ B cells in the bone marrow, indicating a maturation defect [41]. This is likely caused by a noncell-autonomous effect, as bone marrow chimeras, in which wildtype bone marrow was transferred into *Bmal1*-deficient mice, reproduced the phenotype, whereas this was not the case when *Bmal1*-deficient bone marrow was grafted into control mice [41].

At the molecular level, chromatin-immunoprecipitation (ChIP) assays in monocytes and peritoneal macrophages demonstrated that BMAL1, in a heterodimer with CLOCK, binds directly and in a rhythmic manner to E-box motifs within the genes of the chemotactic cytokines *Ccl2*, *Ccl8,* and *S100a8*, exhibiting a peak at *Zeitgeber* time (ZT) 8 [31]. These chemokines are important factors for the chemotaxis and trafficking of these cells and BMAL1:CLOCK binding inhibits their transcription in a time-of-day-dependent manner. This rhythmic repression results in the diurnal expression of these chemokines and is responsible for the observed trafficking dynamics of monocytes such as abundance in blood and migration to organs at specific times of the day [31]. The inhibitory effect is mediated by recruitment of the polycomb repressor complex 2 (PRC2) to the promoter region of the genes via BMAL1, which leads to trimethylation of histone H3 at lysine 27 (H3K27Me3) sites within the *Ccl2*, *Ccl8,* and *S100a8* promoters [31].

Another study demonstrated that also tolllike receptor 9 (TLR9), a pattern recognition receptor for intracellular pathogen-associated molecular patterns, is under direct control of BMAL1:CLOCK. This indicates that BMAL1 activity is an important host immune mechanism in the response to pathogenic environmental factors. Performing ChIP assays in murine macrophages, the authors were able to demonstrate that BMAL1 binds to an E-box motif within the *Tlr9* promoter and upregulates its transcription [37]. Moreover, oscillations in TLR9 levels were also observed in splenic B cells and DCs.

Although the effects of BMAL1 on the immune system are often mediated by its transcriptional activity, it does not always require direct DNA binding. Immunoprecipitation (IP) assays in human embryonic kidney (HEK)-293 cells and mouse embryonic fibroblasts (MEFs) revealed protein-protein interactions between BMAL1 and RelB, a subunit of the proinflammatory transcription factor NFκB, which is a major nexus of the cellular inflammatory response [42]. CLOCK was not required for this interaction; its presence, however, modulated the binding by reinforcing the contact between BMAL1 and RelB, as shown by IP after cotransfection of both clock genes [42]. This indicates an additional and important structural regulatory mechanism of BMAL1 that can influence the immune system on a molecular level, independently of direct transcriptional control.

More insights into the molecular functions of BMAL1 on the immune system were drawn at the organismal levels from conditional BMAL1 deletion models in mice targeting specific immune cell lineages. Most studies have thus far investigated the role of BMAL1 in the myeloid lineage, using *Lyz2*cre:*Bmal1*^flox^ mice. These mice exhibit a lack of rhythm in clock genes in macrophages and greatly reduced expression of clock components such as *Nr1d1* [28,31,[43], [44], [45]]. Lack of myeloid BMAL1 increases the expression of the proinflammatory mediators IL-6, TNF-α, and IL-1β at the mRNA and protein level as shown by Q-PCR and ELISA analyses (Table 1). Mechanistically, loss of myeloid BMAL1 was shown to reduce Nuclear factor erythroid 2-related factor 2 (*Nrf2*), which protects against oxidative damage by driving the expression of antioxidant proteins as demonstrated by Q-PCR [45]. NRF2 is under direct positive control of BMAL1 as shown by transcription factor sequencing. Deficiency in myeloid *Bmal1* abolished the usually antioxidant mechanisms of this protein. Hence, isolated bone-marrow-derived macrophages (BMDMs) exhibit a higher expression of ROS, HIF-1α, IL-6, and IL-1β, indicating an enhanced inflammatory phenotype [45]. This heightened inflammatory state is the likely explanation for the reduced survival observed in *Lyz2cre*:*Bmal1*^*flox*^ mice after the induction of sterile inflammation with lipopolysaccharide (LPS) [28,44,45] or of sepsis [31,43].

**Table 1:** Immune phenotypes in clock-specific global and conditional knockout systems.

Clock gene	Model	Effect	Tissue/cells	Reference
*Bmal1*	KO	Impaired B cell development, reduced B cell numbers Accelerated aging with increased risk for arthropathy	Blood, spleen	[41]
	*Chimera*	*Leishmania* infection: - Loss of time-dependent infection extent - Loss of time-dependent neutrophil and macrophage infiltration - Loss of time-dependent rhythmic chemokine expression	Neutrophils, peritoneal macrophages	[88]
	*Lyz2*-Cre	Reduced survival after LPS	Macrophages	[44]
		Reduced survival after Listeria infection	Monocytes	[31]
		Increased proinflammatory state after LPS:	Macrophages	
		- Increased IL-6		[28]
		- Increased TNFa, reduced *Il10*		[44]
		- Increased ROS, HIF1α, *Il6*, *Il1b*		[45]
		Increased proinflammatory cytokines after *L. monocytogenes* infection	Monocytes	[31]
		Increased proinflammatory miR-155 cluster	Macrophages	[44]
		Higher phospho-p65		
		Increased inflammatory state in diseases:		
		- Allergic asthma	Eosinophils	[46]
		- EAE (increased IM infiltration, increased IL-1β)	CNS	[47]
	*hMRP8*-cre	Impaired clearance of aged neutrophils	Neutrophils	[22]
	*Cd4*-cre	Impeded LN homing Loss of lymph and LN half-life diurnal oscillation	T cells	[27]
	*Cd19*-cre	Loss of oscillations in lymph and LN	B cells	[27]
	*Rorgt-*cre	Reduced small intestinal *lamina propria* (siLP) ILC3s	siLP ILC3	[89,90]
		Reduced epithelial reactivity	Small intestine	
		Increased infection with *Citrobacter rodentium*		
*Clock*	KO	Reduced NFκB activity	Hepatocytes, MEFs	[55]
	Δ19 mutant	Reduced inflammatory cytokines after LPS, TNFα	MEFs	[42]
		Reduced *Il6*, *Il1b*, *Cxcl1*, *Ifnb,* & *Ccl2* after inflammatory stimuli	Macrophages (BMDMs)	[56]
	Δ19 mutant chimera with W/Wv mice	Loss of IgE-mediated degranulation and mast cell signaling	Mast cells	[91]
		Loss of *FcεRIβ* oscillation		
*Per1*	KO	Increased TNFα, IL-1β, IL-6, & MCP-1 after LPS	Macrophages	[57]
*Per2*	KO	Reduced IFNγ, IL-1β after LPS	Plasma	[58]
		Resistant to septic shock		
	Per2-Brdm1	Reduced TNFα, IL-12 after CpG	Macrophages	[37]
		Increased IL-6 after LTA, loss of IL-6 rhythm	Macrophages	[60]
		Loss of diurnal anaphylactic cutaneous reaction	Mast cells (skin, BM)	[61]
*Cry1*	KO	Increased IL-6	Fibroblasts	[64]
*Cry1/2*	KO	Increased T cell cellularity	MEFs, Splenocytes	[63]
		Increased TNFα		
		Increased IL-6, Cxcl1, iNOS, constant NFκB activity	Hypothalamus, Fibroblasts, Macrophages	[64]
		Increased LPS hypersensitivity		
		Spontaneous autoimmune disease:		
		Increased IgG and IgM	Serum, lung, kidney	[65]
		Increased IL-1β, IL-6, MMP-3, & TNFα in arthritis	Serum	
Rev-Erbα	Agonist	Reduced *Il-6*, *Cxcl11*, *Ccl2*, *Cxcl6,* & *Il19*	Macrophages	[28]
	KO	Loss of diurnal LPS response	Macrophages	[64]
		Increased *Cx3cr1* & *Mmp9*		[66]
		Reduced NKp46+ siLP ILC3s	siLP ILC3s	[92]
		Increased DN and CCR6+ siLP ILC3s		
	*Ccsp-Rev-Erbα-DBD*^*m*^	Increased neutrophilic lung inflammation after aerosolized LPS	Lung	[71]
RORα	Staggerer	Increased IL-1β, IL-6, & MIP-2 after LPS	Lung	[72]

Lack of myeloid BMAL1 has been further shown to lead to an increase in the expression of pyruvate kinase muscle isozyme M2 (PKM2), a crucial enzyme in the last step of glycolysis and lactate production. This boosts PD-L1, an antiinflammatory protein that serves as an immune checkpoint for T cell activation, in a STAT1-dependent manner and leads to an increased susceptibility of *Lyz2cre*:*Bmal1*^*flox*^ animals to sepsis-induced death after cecal-ligation and puncture, due to T cell exhaustion and lack of microbial clearance [43].

In line with the structural association of BMAL1 with NFκB components discussed above [42], BMAL1 ablation in myeloid cells was shown to be associated with a rise in the proinflammatory microRNA cluster miR-155 in macrophages. miR-155 directly targets and decreases *Bmal1* RNA transcripts and increases phospho-p65, a subunit of NFκB, as shown by immunoblot analysis [44]. This closely ties together lack of myeloid BMAL1 with increased NFκB activity, resulting in an enhanced inflammatory state.

Since the *Lyz2cre* driver affects all myeloid cells, it is not entirely clear which cell type is mainly responsible for the observed inflammatory phenotypes. Hence, *Mrp8cre:Bmal1*^*flox*^ mice were used in one study to specifically target neutrophils, which shed light on the regulation of neutrophil aging, a process that occurs in a daily manner, and their role in vascular protection and immune defense [22]. This study—using intravital imaging techniques as well as parabiotic mouse models—demonstrated that *Bmal1* regulates the circadian compartmentalization of neutrophils to favor neutrophil migration into multiple tissues during the behavioral active phase while vessels were protected from these highly active cells during the rest phase [22]. Using *Mrp8cre*-specific *Cxcr2* and *Cxcr4* deficient mouse models paired with transcriptome analysis, it was shown that neutrophil aging is induced by *Bmal1* via regulation of *Cxcl2*, which in turn modulates CXCR2 to favor aging while CXCR4 antagonizes this effect [22].

There is now also evidence demonstrating that not only the acute innate immune response is affected by circadian clocks but that also adaptive immunity is clock-controlled. Myeloid BMAL1 deficiency in an ovalbumin-induced model of allergic asthma showed increased eosinophilic infiltration in the lung. In addition, bone-marrow-derived macrophages isolated from *Lyz2cre:Bmal1*^*flox*^ animals and stimulated with LPS *ex vivo* exhibited a higher expression of the asthma-relevant chemokines CCL2, CXCL10, as well as the asthma-associated mannose receptor [46]. Interestingly, a higher disease severity was observed in wildtype mice in the late afternoon compared to other time points, weeks after induction with myelin oligodendrocyte glycoprotein (MOG) to induce experimental autoimmune encephalomyelitis (EAE), a mouse model of multiple sclerosis [47]. In myeloid BMAL1-deficient animals, in contrast, this time-of-day dependency was lost and disease scores were higher compared to wildtype mice [47]. Loss of myeloid *Bmal1* in EAE increased the proinflammatory cytokines IL-12p40 in serum as well as IL-1β and IL-23 in GM–CSF–expanded bone marrow cells in response to *Mycobacterium tuberculosis* [47]*.* In addition, MOG-induced *Lyz2cre*:*Bmal1*^*flox*^ mice exhibited higher Th1 and Th17 cell responses in the CNS compared to control animals and *ex vivo*-expanded bone marrow cells from these animals showed increased IFN-γ levels in coculture with MOG-specific wildtype CD4^+^ T cells [47]. This demonstrates an extended influence and pre-conditioning effects of lack of myeloid *Bmal1* on other cell types, specifically also those of the adaptive arm of immunity [43,47].

Concerning the role of BMAL1 in lymphocytes, a study using B cell (*Cd19cre:Bmal1*^*flox*^) and T cell (*Cd4cre:Bmal1*^*flox*^)-specific *Bmal1*-deficient mouse models highlighted that expression of this gene in lymphocytes themselves is dispensable for their development [48]. This is in line with the previously mentioned report that BMAL1 is important for B cell development only in a noncell-autonomous manner [41]. Specifically, no difference in cellularity or phenotype was observed in the thymus of T cell-specific BMAL1-ablated animals versus control animals. However, the authors detected a reduction of BMAL1-deficient CD8 T cells compared to cotransferred wildtype cells into the same, *L. monocytogenes*-infected host in their production of IL-2, IFN-γ, and TNF-α [48]. Furthermore, T cells lacking *Bmal1* were impeded in their rhythmic homing capacity to the lymph node as well as in their lymph node transition time [27]. *Cd4cre*:*Bmal1*^*flox*^ mice, analogous to *Lyz2cre:Bmal1*^*flox*^ mice, furthermore failed to show a time-of-day difference in EAE disease symptoms, with, however, an overall reduced disease score [27]. While wildtype animals displayed a significantly heightened disease severity after EAE induction at ZT8 compared to ZT20, *Cd4*cre:*Bmal1*^*flox*^ mice lost this time-dependent effect and EAE scores were comparable with the low disease severity associated with immunization at ZT20 [27]. In *Cd19cre:Bmal1*^*flox*^ mice, B cells lost their oscillatory abundance in lymph, implying a role of BMAL1 also in lymph node egress of lymphocytes [27]. This is in line with a study that used β2 adrenergic receptor (*Adrb2*) deficient mice to show that *Adrb2* expression in B cells is necessary for their rhythmic retention in the lymph node [49] potentially via direct binding to CXCR4 as shown by IP experiments [50].

CXCR4 is a critical chemokine receptor for rhythmic leukocyte migration [21,51]. Diurnal rhythms in CXCR4 protein and mRNA expression levels were observed to be lost in CD4 and CD8 T cells in T cell-specific glucocorticoid-receptor (GR)-deficient animals (*Cd4cre*:*Nr3c1*^*flox*^) [52]. This impeded migratory rhythms of T cells in *in vitro* transwell migration assays in response to the CXCR4 ligand CXCL12 as well as rhythmic trafficking in blood, lymph node, and spleen. Mechanistically, the glucocorticoid receptor was shown by ChIP assays to rhythmically bind to enhancer elements in the IL-7Ra locus, inducing IL-7R expression and—in turn—expression of CXCR4 [52].

Moreover, lack of *Bmal1* in mature CD8 T cells abolished circadian CD8 T cell responses to antigen presentation by DCs. This was demonstrated by injection of LPS-stimulated bone-marrow-derived DCs, loaded with OVA-peptide (OVA-DC), into control and CD8 T cell-specific *E8Icre:Bmal1*^*flox*^ animals during day and night [33]. While control animals displayed increased OVA-specific CD8 T cells in spleen as determined by K^b^-OVA tetramer staining as well as higher CD8^+^CD44^+^IFNγ^+^ abundancies after *ex vivo* restimulation with OVA peptide upon OVA-DC injection during daytime [35], this effect disappeared in animals lacking *Bmal1* in CD8 T cells [33]. Using RNA sequencing analyses, the authors demonstrated that CD8^+^ T cells are prone to respond more strongly during the daytime due to increased expression of TCR-dependent signaling pathways as well as genes related to elevated T cell activation and proliferation [33].

Together, these studies indicate an important, time-specific role for lymphocyte BMAL1 in adaptive immunity, mediated by directly affecting lymphocyte function and indirectly by governing lymphocyte trafficking behavior.

## CLOCK

The expression of CLOCK is important for maintaining circadian behavior since *Clock* mutant mice (*Clock*Δ19, a point mutation that leads to loss of exon 19) exhibit a lengthened circadian period and can lose their rhythmic locomotor activity in constant darkness as assessed by wheel-running activity [53]. While this mutation leads to a transcriptionally near inactive BMAL1:CLOCK heterodimer, *Clock*^*−/−*^ animals maintain their circadian behavior in constant darkness, likely due to rescue by the CLOCK paralog neuronal PAS domain protein 2 (NPAS2). In addition to its heterodimerization with BMAL1, the transcriptional role is also mediated by the intrinsic HAT activity of CLOCK. Recruitment of CLOCK to DNA induces the acetylation of lysine residues in histones H3 and H4, which leads to DNA opening and facilitated transcription. This function is crucial for circadian rhythmicity, since ectopic expression of a HAT-deficient CLOCK mutant (mCLOCK-mut A) was not able to restore circadian rhythms in a *Clock*^−/-^ MEF cell line, established from homozygous *Clock* mutant mice [18]. The HAT activity of CLOCK is essential for reducing transcriptional activity of the GR [54]. CLOCK was demonstrated to exhibit a physical interaction with the GR (using IP assays), which resulted in the suppression of GR DNA-binding capacity by CLOCK-mediated acetylation of lysine-residues within GR. This caused a spontaneous, reversed-phase circadian fluctuation in GR activity *in vitro*, as demonstrated by mRNA expression of GR response genes [54].

Similar to BMAL1, CLOCK has also been shown to directly influence proinflammatory proteins. One study identified CLOCK to be situated in a protein complex together with the NFκB subunit p65 (RelA), using co-IP assays [55]. This interaction was counteracted by BMAL1, which recruited CLOCK into the heterodimer and thus reduced the amount of free CLOCK protein. *Clock*^*−/−*^ mice exhibited reduced nuclear NFκB accumulation in MEFs and primary hepatocytes, shown by immunostaining as well as *in vivo* imaging in an IκB-Luciferase (IκBα-Luc), *Clock*-deficient reporter mouse strain. This reduced NFκB activity was assessed by cotransfections of the κB-Luc reporter plasmid together with combinations of p65-, CLOCK-, and BMAL1-expressing plasmids. In contrast, *Clock*Δ19 mutants did not show reduced activation of NFκB responsive genes. This points toward a mechanism that is not dependent on CLOCK-mediated transcription, as the *Clock*Δ19 mutation allows BMAL1 heterodimerization and DNA binding but fails to activate transcription [55].

Moreover, an interaction of CLOCK with other NFκB coactivators, such as CREB binding protein (CBP) was demonstrated by cotransfections of κB-Luc expressing HEK cells with combinations of CBP-, CLOCK-, or BMAL1-expressing plasmids. Coexpression of CLOCK and CBP resulted in higher NFκB activation [55]. These data are in line with other studies showing a proinflammatory role of CLOCK. However, reduced proinflammatory cytokine expression of *Il6*, *Cxcl1,* and *Il1b* mRNA levels were seen in MEFs or BMDMs harvested from *Clock*Δ19 mice after LPS or TNF-α stimulation, thus implicating reduced transactivation activity in the process [56]. Interestingly, these clock mutant MEFs displayed overexpression of the negative NFκB pathway element RelB after LPS treatment as demonstrated in immunoblots [42]. The interaction with the NFκB-subunit RelB was shown to be mainly dependent on BMAL1 as cotransfection of CLOCK and RelB by themselves did not lead to co-IP. However, a functional CLOCK protein was required for the transcriptional activity as demonstrated by analyzing the induction of the target gene *Per1* in transiently transfected luciferase assays [42].

Together, these data paint a picture of a proinflammatory role of CLOCK that mediates its effects via direct interactions with components of the NFκB pathway but also via its transactivational role in a complex with BMAL1.

## PER Proteins

The role of PER proteins in the immune system has been investigated *in vitro* using primary macrophages harvested from blood and the peritoneal cavity, as well as with the RAW264.7 monocytic cell line, and *in vivo* using *Per1* and *Per2* single and double deficient mice. Furthermore, the mouse PER2::Luc reporter line has enabled and facilitated research on the clock and the role of *Per2* in particular. Additionally, *Per1/2* mutant mice have been generated, which exhibit a defective circadian clock. *Per1*^*−/−*^ mice exhibit increased expression of inflammatory cytokines such as TNF-α, IL-1β, IL-6, and CCL2 in serum, as well as increased mRNA levels of these cytokines in the liver after injection of LPS and D-galactosamine, a hepatotoxicant [57]. This results in a higher susceptibility of these mice to LPS challenge and ensuing decreased survival rates, which are likely due to acute liver failure, as increased serum levels in the hepatocyte-specific enzymes alanine transaminase and aspartate transaminase were observed [57].

In contrast, PER1 regulates expression of the inflammatory chemokine receptor CCR2 in macrophages together with the nuclear receptor peroxisome proliferator-activated receptor gamma (PPARγ) by binding to the *Ccr2* promoter, as analyzed using ChIP analyses [57]. In *Per2*^−/−^ mice, the oscillation in inflammatory markers usually observed in wildtype mice after LPS challenge was abolished, but—in contrast to *Per1*^*−/−*^ mice—IFN-γ and IL-1β levels in plasma were strongly reduced at all circadian time points and amounts of TNF-α, IL-6, and IL-10 were not affected [58]. Similarly, in the *Per2* mutant (*Per2*^*mut*^) the usually observed daily rhythms in IFN-γ mRNA and protein levels in spleen and serum were lost [59]. *Per2*^*mut*^ mice additionally display a loss of *Tlr9* mRNA oscillation and decreased TLR9-dependent CpG-induced cytokine responses in macrophages as shown by their significantly lowered TNF-α and IL-12 production compared to wildtype cells [37]. *Per2*^*mut*^ mice lost the usually observed diurnal oscillations in disease severity of sepsis (caused by cecal ligation and puncture) [60]. This effect was dependent on PER2 expression in radiosensitive hematopoietic cells as *Per2*^*mut*^ bone marrow transplants into wildtype reproduced the phenotype but not when wildtype was transplanted into *Per2*^*mut*^ recipients [60]. *Per2* mutants furthermore lose the time-of-day dependence in cutaneous anaphylactic reactions by deregulation of the responsiveness of mast cells to corticosterone [61]. The upregulation of serum corticosterone levels during the day coincides with high PER2 expression levels in mast cells (shown using *Per2:Luc* mice), which allows transduction of corticosterone signaling. Together, these data indicate that the role of PER proteins in the immune system is complex, as PER1 has been mostly associated with antiinflammatory effects, while PER2 has been suggested to work as a positive regulator of proinflammatory cytokines. A possible explanation for this divergence lies in the fact that although both molecules are direct targets of GRα, only PER2 was shown to form physical complexes with nuclear receptors [62].

## CRY Proteins

Similarly to the PER proteins, the role of CRY proteins in the immune system has been primarily investigated in cell culture (using fibroblasts and 293T cells) and *in vivo* using *Cry1*^−/−^ *and Cry2*^−/−^ single and double deficient mice. *Cry1*^−/−^*Cry2*^−/−^ mice exhibit an elevated T cell cellularity in the spleen with increased TNF-α levels [63]. In a collagen type II mouse experimental arthritis model, *Cry1*^−/−^*Cry2*^−/−^ mice displayed aggravated pathological changes in the arthritis disease score and increased serum levels of IL-1β, IL-6, MMP-3, and TNF-α as well as c-FOS and Wee-1 protein levels in spleen, markers also upregulated in human arthritis. This increase could be reduced in MEFs from *Cry1*^−/−^*Cry2*^−/−^ mice upon ectopic reexpression of *Cry1* [63]. Another study showed that in *Cry1*^−/−^*Cry2*^−/−^ mice a high constitutive level of the proinflammatory cytokines IL-6, TNF-α, and iNOS were expressed in the hypothalamus [64]. Furthermore, BMDMs from *Cry1*^−/−^*Cry2*^−/−^ mice showed a marked increase in expression of inflammatory cytokines such as *Il6*, *Cxcl1*, and *Nos2* and a hypersensitivity to LPS stimulation [64]. Mechanistically, it was shown that the NFκB signaling pathway was constitutively activated in *Cry1*^−/−^*Cry2*^−/−^ BMDMs, concomitant with continuous transcription of IL-6 and reduced expression of cAMP [64]. This was shown by IP to be due to direct binding of CRY1 to the adenylyl cyclase, inhibiting adenylyl cyclase function and thus limiting the PKA-induced phosphorylation of p65, thereby inhibiting NFκB activation. Due to the ensuing higher constitutive inflammatory state, *Cry1*^−/−^*Cry2*^−/−^ mice spontaneously manifest autoimmune-like diseases. They exhibit higher serum antinuclear IgG antibody levels (rendering them susceptible to autoimmune diseases), increased glomerular deposits of IgG and IgM antibodies, and complement C3 and strong infiltration of leukocytes in lungs and kidneys [65]. To conclude, CRYs are important antiinflammatory proteins that control the intensity of immune responses by downregulating inflammatory cytokines.

## REV-ERB

As with PER and CRY, there is compelling evidence for a role for REV-ERBα (encoded by *Nr1d1*) and RORα in the control of the immune system. In addition to their *bona fide* role as circadian clock components, REV-ERB proteins play a pivotal role as output mediators of the clock. As such, REV-ERBα acts as a handle for the clock to regulate immunity in many contexts, primarily exerting an antiinflammatory role.

The synthetic REV-ERBα ligand GSK4112 was shown to reduce IL-6 release from human blood macrophages and to decrease mRNA levels of the proinflammatory cytokines and chemokines *Cxcl11*, *Ccl2*, *Cxcl6,* and *Il19* during LPS challenge [28]. Moreover, *in vivo* challenge of *Nr1d1*^−/−^ mice with LPS lead to loss of rhythmic IL-6 upregulation in serum in comparison to wildtype animals [28]. Analysis of *Nr1d1*^−/−^ BMDMs in steady state showed increased mRNA levels of *Cx3cr1* and *Mmp9*, whereas overexpression of REV-ERBα in this cell type decreased these mRNA levels [66]. Mechanistically, it was shown by IP in macrophages that REV-ERBα is able to bind to histone deacetylase 3 (HDAC3) and recruit a repressor complex consisting of HDAC3 and the nuclear hormone corepressor (NCoR) to the DNA to negatively regulate gene expression of cytokines such as *Il6* [66]. REV-ERBα has also been shown to be a negative regulator of the NLRP3 inflammasome by binding to the promoter region of *Nlrp3* and *Il1b* and was additionally shown to be able to regulate *Il18* as observed by ChIP sequencing of promoter regions [67].

Moreover, REV-ERBα additionally directly binds to Rev responsive elements in the p65 promoter as demonstrated by luciferase reporter assay, Electrophoretic Mobility Shift Assay (EMSA), and ChIP sequencing in Raw264.7 cells. Treatment with the REV-ERBα agonist SR9009 reduced total, cytosolic, and nuclear p65 levels. The same study also highlighted the role of REV-ERBα in repressing Nlrp3 inflammasome activation by directly repressing *Nlrp3* transcription as shown in luciferase reporter assays, EMSA, and ChIP sequencing in Raw264.7 and HEK293 cells, as well as in peritoneal macrophages and in colon [68]. Together, this repression of the NFκB/Nlrp3 axis provides a possible mechanism on how the antiinflammatory role of REV-ERBα is mediated.

Using *Nr1d1*-deficient mice and human macrophages, it was shown that NLRP3 expression and activation of its complex varied according to time of day, peaking in the late night, which was negatively dependent on *Nr1d1* expression. Activation of REV-ERBα was shown to inhibit the NLRP3 inflammasome pathway in acute LPS-induced peritonitis and hepatitis [67]. In addition, REV-ERBα was demonstrated to regulate CCL2, thus mediating the circadian sensitivity to viral infections in a vesicular stomatitis virus (VSV)-induced encephalitis model [69]. Specifically in macrophages, REV-ERBα downregulates *Ccl2* expression by binding to a proximal ROR element in the murine *Ccl2* promoter—as shown by ChiP assays—inhibiting expression [70]. Depletion of the REV-ERBα DNA binding domain (DBD) in bronchoepithelial cells using *Ccsp-Rev-Erbα-DBD*^*m*^ mice in a PER2::luc background demonstrated a markedly increased neutrophilic response to aerosolized LPS in bronchoalveolar lavage fluid while circadian PER2 luminescence in bronchioles was not altered [71]. This indicates another antiinflammatory role of REV-ERBα, dependent on its DBD, while circadian regulation seemed not to be affected by this mutation.

In contrast to the generally antiinflammatory effects of REV-ERBα stated above, REV-ERBα was also shown to drive the differentiation of the inflammatory Th17 immune subset by directly repressing *Nfil3* transcription due to its binding to a consensus sequence in the *Nfil3* gene locus as shown by ChIP [17]. Loss of REV-ERBα was shown to yield higher expression of *Nfil3* in CD3^+^ T cells, which in turn enabled RORγ T cells to induce IL17 production and thus induce the development of Th17 cells [17]. Altogether, however, the antiinflammatory effect of REV-ERBα dominates, mediated via its inhibition of the inflammasome.

## ROR

RORα opposes REV-ERBα functions as it increases the promoter activity of *Ccl2* as shown in promoter binding assays [70]. Mice deficient in RORα (*Rora*^*−/−*^*,* also known as the staggerer mutant, RORα^sg/sg^) present a severe ataxic neuronal phenotype [72]. They also exhibit higher levels of IL-1β, IL-6, and MIP-2 in bronchoalveolar lavage (BAL) fluid, which renders them more susceptible to LPS lethality after intratracheal instillation [72]. RORα plays a critical role in lymphocyte development as *Rora*^*−/−*^ mice have reduced splenic and thymic cellularity [73]. This is due to severely defective development in the B- and T cell compartment, which is caused by a noncell-autonomous effect [73], analogous to what has been observed for BMAL1 [41]. The CD8 T cells that do exist exhibit increased IFN-γ production after T cell receptor stimulation and elevated IgG levels after immunization with T cell dependent antigens [73]. Interestingly, *Rora*^*−/−*^ mast cells and macrophages—but not T cells—exhibit an increased expression of TNF-α and IL-6, indicating that RORα acts as a negative regulator of inflammatory cytokines in a subset-specific manner [73].

In macrophages, RORα maintains a resting, nonactivated state, which prevents an early innate immune response, similar to CD8 T cells [73]. RORα was furthermore shown to bind to ROR elements in the promoter region of *IκBα* (nuclear factor of kappa light polypeptide gene enhancer in B cells inhibitor alpha), thus upregulating IκBα transcription, which is a major inhibitor of the NFκB signaling pathway [74]. This leads to reduced nuclear translocation of p65 and reduced transcription of target genes [74]. As a consequence, RORα negatively regulates the cytokine-induced inflammatory response [74].

To conclude, RORα and REV-ERBα are closely regulated by BMAL1 and provide feedback to induce or repress BMAL1 expression, respectively, via ROR elements in the BMAL1 gene. Directly, but also via REV-ERBα and RORα, BMAL1 is able to act in an antiinflammatory manner due to an upregulation of, for example, the NFκB signaling pathway. Although REV-ERBα and RORα exhibit antagonizing functions with respect to their role on BMAL1 expression, both exhibit antiinflammatory functions in the immune system.

## Influence of the Immune System on Clock Proteins

Interactions between clock proteins and immunity are also taking place in a reciprocal manner (Table 2). Inflammation reduces behavioral locomotor activity and dampens behavioral oscillations [75]. Continuous subcutaneous application of TNF-α in mice using osmotic minipumps for three days abolished locomotor activity accompanied by prolonged rest time [75]. Furthermore, a significant decrease of the clock-controlled gene *Dbp* was observed in the SCN of these mice [75].

**Table 2:** Clock and circadian phenotypes upon modulation of the immune system.

Immune stimulus	Effect	Tissue/cells	Reference
LPS	Delays in circadian activity		[77,78]
	Disrupted clock gene expression:		
	- Reduced *Dbp* and *Per2*	SCN	[76]
	- Increased *Per1*	PVN	[64]
	- *Per2* phase shift, reduced *Bmal1*	Macrophages	[57]
	- Reduced *Per1* and *Per2*	Heart, liver	[85]
	- Altered *Dbp*, *Ppara*, *Fkbp51*	Liver	[76]
	- Reduced *Per2*	Ovaries	[86]
	- Disrupted rhythms and new rhythms	Lung	[87]
	- Reduced *Bmal1*	Macrophages	[44]
TNFα	Reduced LMA, prolonged rest phase		[75]
	Altered spiking activity	SCN	[80]
	Reduced *Dbp*	SCN	[75]
	Reduced *Per1-3*, *Dbp*, *Tef*, *Hlf*	Fibroblasts	[75]
	Increased CRY1, reduced *Dbp*, *Per2*	Hepatocytes/liver	[81]
IFNγ	Altered spiking activity	SCN	[80]
	Reduced *Per1*	SCN	[80]
IFNα	Reduced BMAL1 and CLOCK	Hepatocytes/liver	[82]
Salmonella	Reduced *Per2*	Macrophages	[56] [79]
Turpentine oil	Reduced *Per1* and *Per2* peaks	Liver	[93]
	Shifted *Per2* oscillation	Heart	

Peripheral injections of LPS into the peritoneal cavity transiently suppressed *Per2* and *Dbp* mRNA expression levels and reduced amplitudes of clock genes in the SCN of rats [76]. These changes on expression levels of circadian clock genes in the central clock might explain transient alterations such as abolished locomotor activity for three days and a −40min phase delay in circadian activity upon sublethal or low-dose LPS administration, respectively [77,78]. Interestingly, administration of the NFκB inhibitor sulfasalazine was able to suppress this phase-delay, implying a direct influence of this key immune transcription factor on the regulation of the central circadian clock [77]. This supports the close functional associations between members of this signaling pathway and clock components, discussed above. Nevertheless, all studies have shown that interruption was only temporary, for a maximum of three days, indicating that—while oscillations are dampened—the central clock still remains entrained to the environment during an immune response.

Mechanistically, it was demonstrated that LPS injections induced cellular activation in the SCN by measuring increased c-FOS and p65-NFκB expression levels in SCN neurons [79]. Inflammatory mediators can act directly on the SCN as diurnal expression levels of IL-1R1 have been observed in the SCN of mice [79]. In line with these data, adding IFN-γ on SCN cells grown in culture reduced the amplitude of *Per1*-luciferase rhythms and decreased spontaneous excitatory and altered spiking activity [80]. These studies hence indicate a direct molecular effect of immune factors on the central clock. However, *in vivo* the interplay between bacterial products acting directly on the SCN and indirectly via inflammatory mediators released by the immune system is not clear.

In the periphery, LPS administration can disrupt clock gene expression in multiple cell types and organs. Similar to the effect of TNF-α on altering *Dbp* expression in SCN tissue sections as shown by *in situ* hybridization, this cytokine exhibited an even more profound effect on clock genes in cultured fibroblasts by reducing expression of *Per1*, *2,* and *3*, as well as diminishing amplitudes of the clock-controlled genes *Dbp*, *Tef,* and *Hlf*. TNF-α interferes with the expression of E-box containing clock genes as demonstrated by stably transfecting NIH3T3 cells with a luciferase reporter plasmid consisting of three E-boxes and measuring luciferase activity after TNF-α treatment [75]. In contrast, TNF-α increased CRY1 protein levels in cultured mouse hepatocytes, which was mediated by activation of USP2a, a circadian controlled deubiquitinating enzyme, which in turn stabilized CRY1 protein as shown by reduced ubiquitination status using IP [81]. After i.v. injection of adenoviral vectors containing shRNA targeting *Usp2a in vivo* a downregulation of *Dbp* and *Per2* in the liver was observed [81]. Administration of IFN-α was shown to reduce protein levels of CLOCK and BMAL1 in cultured hepatocytes, as well as in mouse liver [82]. This reduction led to blunted rhythms in clock and clock-controlled genes such as *Per1* and *Dbp* as demonstrated by Q-PCR. Mechanistically, this was shown to be STAT1-dependent since application of the IFN-α-inhibitor aurintricarboxylic acid significantly reduced elevated phospho-STAT1 levels, which was accompanied by the restoration of *Clock* and *Bmal1* mRNA levels [82]. Isolated peritoneal macrophages displayed a clear *Per2* phase shift and reduction in *Bmal1* expression after culture with low doses of LPS. This effect was dependent on TLR4 as shown using hypoacylated LPS (LPS-RS) as competitive antagonist for TLR4, as well as on ROS production, since LPS-RS as well as a NOX2 inhibitor and superoxide dismutase reversed the LPS-induced circadian disruption [83]. Treating bone-marrow-derived macrophages *in vitro* with *Salmonella typhimurium* altered rhythmicity in circadian and metabolic gene expression with decreased *Per2* mRNA levels [56].

Moreover, the administration of molecules exhibiting pathogen associated molecular patterns (PAMP), such as the TLR3 agonist polyinosinic:polycytidylic acid (poly I:C) altered clock gene expression in an *ex vivo* study using splenocytes, indicating a major influence of infectious stimuli on the circadian clock [84]. On a mechanistic level, a direct influence of the NFκB subunit RelB on the activity of the BMAL1-CLOCK heterodimer was described as demonstrated by reduced luciferase-fused E-box transactivation oscillation upon ectopic expression of RelB. In this study, it was shown that RelB significantly suppressed BMAL1-CLOCK transcriptional activity at *Dbp* and *Per1* promoters by a direct interaction with BMAL1 in the presence of CLOCK by using ChIP sequence. Additionally, RelB was observed to control the amplitude of circadian transcription since RelB-deficient fibroblasts displayed increased amplitudes of *Dbp*, *Nr1d1,* and *Cry1* but decreased *Per* expression [42].

*In vivo*, heart and liver tissue showed decreased *Per1* and *Per2* expression levels in a time-dependent manner after LPS administration [85]. Another study recorded altered expression levels of clock-controlled genes *Dbp*, *Ppara,* and *Fkbp51* in the liver one day after LPS injection with a full recovery on the second day [76]. Reduced *Per2* amplitude was also found in ovaries after LPS administration [86] and disrupted rhythmic gene expression was observed in a lung inflammatory model [87]. Here, the authors even observed induction of new rhythms such as in adenine and 21 other metabolites [87]. Moreover, a rapid loss of REV-ERBα protein in inflamed lung tissue was detected after the inhalation of aerosolized LPS, which could be mimicked *in vitro* by administration of TNF-α and IL-1β in synchronized lung epithelial cells [71].

Although these studies used different inflammatory stimuli as well as target cell types and tissues, they all demonstrated a clear disruption of circadian rhythmicity in peripheral clocks, indicating a profound influence of the immune system on clock gene regulation.

These data enforce the importance of the immune system in influencing circadian activity by directly modulating clock gene expression.

## Outlook

Many studies have shown a circadian rhythm in immune responses. Since the initial descriptive studies, it has become increasingly clear in the last years that there is a bidirectional molecular relationship between clock proteins and components of the immune system. BMAL1 exhibits mostly antiinflammatory roles, while its heterodimerization partner CLOCK rather activates the immune system. PER proteins can act in both ways, amongst others, by modulating expression of BMAL1 while CRY proteins, REV-ERB, and ROR present generally antiinflammatory functions. Clock proteins are transcription factors and therefore influence gene transcription directly, as well as by recruiting either activating or repressing enzymes to the promoter region of immune-associated genes. Additionally, they also physically interact with inflammatory molecules. A key mechanism involves regulation of the inflammatory NFκB pathway, since several publications could demonstrate direct links between clock proteins and NFκB components. Thus, clock proteins form a sensitive network, which strongly controls immune responses. Disruption as shown using several knockout models can lead to severe disease manifestation and immune pathologies. Further studies using cell-type-specific clock-deficient models paired with experiments using clock proteins exhibiting mutated domains will be needed to link specific immune-modulatory functions of a clock protein domain to an immune phenotype.

## References

[1] Woelfle M.A., Ouyang Y., Phanvijhitsiri K., Johnson C.H. (2004). The adaptive value of circadian clocks: an experimental assessment in cyanobacteria. Curr. Biol..

[2] Menet J.S., Rodriguez J., Abruzzi K.C., Rosbash M. (2012). Nascent-Seq reveals novel features of mouse circadian transcriptional regulation. Elife.

[3] Panda S., Antoch M.P., Miller B.H., Su A.I., Schook A.B., Straume M. (2002). Coordinated transcription of key pathways in the mouse by the circadian clock. Cell.

[4] Storch K.F., Lipan O., Leykin I., Viswanathan N., Davis F.C., Wong W.H. (2002). Extensive and divergent circadian gene expression in liver and heart. Nature.

[5] Bell-Pedersen D., Cassone V.M., Earnest D.J., Golden S.S., Hardin P.E., Thomas T.L. (2005). Circadian rhythms from multiple oscillators: lessons from diverse organisms. Nat. Rev. Genet..

[6] Dibner C., Schibler U., Albrecht U. (2010). The mammalian circadian timing system: organization and coordination of central and peripheral clocks. Annu. Rev. Physiol..

[7] Stokkan K.A., Yamazaki S., Tei H., Sakaki Y., Menaker M. (2001). Entrainment of the circadian clock in the liver by feeding. Science.

[8] Damiola F., Le Minh N., Preitner N., Kornmann B., Fleury-Olela F., Schibler U. (2000). Restricted feeding uncouples circadian oscillators in peripheral tissues from the central pacemaker in the suprachiasmatic nucleus. Genes Dev..

[9] Ko C.H., Takahashi J.S. (2006). Molecular components of the mammalian circadian clock. Hum. Mol. Genet..

[10] Huang N., Chelliah Y., Shan Y., Taylor C.A., Yoo S.H., Partch C. (2012). Crystal structure of the heterodimeric CLOCK:BMAL1 transcriptional activator complex. Science.

[11] Busino L., Bassermann F., Maiolica A., Lee C., Nolan P.M., Godinho S.I. (2007). SCFFbxl3 controls the oscillation of the circadian clock by directing the degradation of cryptochrome proteins. Science.

[12] Preitner N., Damiola F., Lopez-Molina L., Zakany J., Duboule D., Albrecht U. (2002). The orphan nuclear receptor REV-ERBalpha controls circadian transcription within the positive limb of the mammalian circadian oscillator. Cell.

[13] Sato T.K., Panda S., Miraglia L.J., Reyes T.M., Rudic R.D., McNamara P. (2004). A functional genomics strategy reveals Rora as a component of the mammalian circadian clock. Neuron.

[14] Takahashi J.S. (2017). Transcriptional architecture of the mammalian circadian clock. Nat. Rev. Genet..

[15] Cho H., Zhao X., Hatori M., Yu R.T., Barish G.D., Lam M.T. (2012). Regulation of circadian behaviour and metabolism by REV-ERB-alpha and REV-ERB-beta. Nature.

[16] Seillet C., Rankin L.C., Groom J.R., Mielke L.A., Tellier J., Chopin M. (2014). Nfil3 is required for the development of all innate lymphoid cell subsets. J. Exp. Med..

[17] Yu X., Rollins D., Ruhn K.A., Stubblefield J.J., Green C.B., Kashiwada M. (2013). TH17 cell differentiation is regulated by the circadian clock. Science.

[18] Doi M., Hirayama J., Sassone-Corsi P. (2006). Circadian regulator CLOCK is a histone acetyltransferase. Cell.

[19] Aguilar-Arnal L., Sassone-Corsi P. (2013). The circadian epigenome: how metabolism talks to chromatin remodeling. Curr. Opin. Cell Biol..

[20] Feng D., Lazar M.A. (2012). Clocks, metabolism, and the epigenome. Mol. Cell.

[21] Zhao Y., Liu M., Chan X.Y., Tan S.Y., Subramaniam S., Fan Y. (2017). Uncovering the mystery of opposite circadian rhythms between mouse and human leukocytes in humanized mice. Blood.

[22] Adrover J.M., Del Fresno C., Crainiciuc G., Cuartero M.I., Casanova-Acebes M., Weiss L.A. (2019). A neutrophil timer coordinates immune defense and vascular protection. Immunity.

[23] Arjona A., Sarkar D.K. (2005). Circadian oscillations of clock genes, cytolytic factors, and cytokines in rat NK cells. J. Immunol. (Baltimore, Md : 1950).

[24] Baumann A., Gonnenwein S., Bischoff S.C., Sherman H., Chapnik N., Froy O. (2013). The circadian clock is functional in eosinophils and mast cells. Immunology.

[25] Boivin D.B., James F.O., Wu A., Cho-Park P.F., Xiong H., Sun Z.S. (2003). Circadian clock genes oscillate in human peripheral blood mononuclear cells. Blood.

[26] Bollinger T., Leutz A., Leliavski A., Skrum L., Kovac J., Bonacina L. (2011). Circadian clocks in mouse and human CD4+ T cells. PLoS One.

[27] Druzd D., Matveeva O., Ince L., Harrison U., He W., Schmal C. (2017). Lymphocyte circadian clocks control lymph node trafficking and adaptive immune responses. Immunity.

[28] Gibbs J.E., Blaikley J., Beesley S., Matthews L., Simpson K.D., Boyce S.H. (2012). The nuclear receptor REV-ERBalpha mediates circadian regulation of innate immunity through selective regulation of inflammatory cytokines. Proc. Natl. Acad. Sci. U. S. A..

[29] Hayashi M., Shimba S., Tezuka M. (2007). Characterization of the molecular clock in mouse peritoneal macrophages. Biol. Pharm. Bull..

[30] Keller M., Mazuch J., Abraham U., Eom G.D., Herzog E.D., Volk H.D. (2009). A circadian clock in macrophages controls inflammatory immune responses. Proc. Natl. Acad. Sci. U. S. A..

[31] Nguyen K.D., Fentress S.J., Qiu Y., Yun K., Cox J.S., Chawla A. (2013). Circadian gene Bmal1 regulates diurnal oscillations of Ly6C(hi) inflammatory monocytes. Science.

[32] Silver A.C., Arjona A., Hughes M.E., Nitabach M.N., Fikrig E. (2012). Circadian expression of clock genes in mouse macrophages, dendritic cells, and B cells. Brain Behav. Immun..

[33] Nobis C.C., Dubeau Laramee G., Kervezee L., Maurice De Sousa D., Labrecque N., Cermakian N. (2019). The circadian clock of CD8 T cells modulates their early response to vaccination and the rhythmicity of related signaling pathways. Proc. Natl. Acad. Sci. U. S. A..

[34] de Juan A., Ince L.M., Pick R., Chen C.S., Molica F., Zuchtriegel G. (2019). Artery-associated sympathetic innervation drives rhythmic vascular inflammation of arteries and veins. Circulation.

[35] Fortier E.E., Rooney J., Dardente H., Hardy M.P., Labrecque N., Cermakian N. (2011). Circadian variation of the response of T cells to antigen. J. Immunol. (Baltimore, Md : 1950).

[36] Gibbs J., Ince L., Matthews L., Mei J., Bell T., Yang N. (2014). An epithelial circadian clock controls pulmonary inflammation and glucocorticoid action. Nat. Med..

[37] Silver A.C., Arjona A., Walker W.E., Fikrig E. (2012). The circadian clock controls toll-like receptor 9-mediated innate and adaptive immunity. Immunity.

[38] Winter C., Silvestre-Roig C., Ortega-Gomez A., Lemnitzer P., Poelman H., Schumski A. (2018). Chrono-pharmacological targeting of the CCL2-CCR2 Axis Ameliorates atherosclerosis. Cell Metabol..

[39] Bunger M.K., Wilsbacher L.D., Moran S.M., Clendenin C., Radcliffe L.A., Hogenesch J.B. (2000). Mop3 is an essential component of the master circadian pacemaker in mammals. Cell.

[40] Bunger M.K., Walisser J.A., Sullivan R., Manley P.A., Moran S.M., Kalscheur V.L. (2005). Progressive arthropathy in mice with a targeted disruption of the Mop3/Bmal-1 locus. Genesis.

[41] Sun Y., Yang Z., Niu Z., Peng J., Li Q., Xiong W. (2006). MOP3, a component of the molecular clock, regulates the development of B cells. Immunology.

[42] Bellet M.M., Zocchi L., Sassone-Corsi P. (2012). The RelB subunit of NFkappaB acts as a negative regulator of circadian gene expression. Cell Cycle.

[43] Deng W., Zhu S., Zeng L., Liu J., Kang R., Yang M. (2018). The circadian clock controls immune checkpoint pathway in sepsis. Cell Rep..

[44] Curtis A.M., Fagundes C.T., Yang G., Palsson-McDermott E.M., Wochal P., McGettrick A.F. (2015). Circadian control of innate immunity in macrophages by miR-155 targeting Bmal1. Proc. Natl. Acad. Sci. U. S. A..

[45] JO Early, Menon D., Wyse C.A., Cervantes-Silva M.P., Zaslona Z., Carroll R.G. (2018). Circadian clock protein BMAL1 regulates IL-1beta in macrophages via NRF2. Proc. Natl. Acad. Sci. U. S. A..

[46] Zaslona Z., Case S., JO Early, Lalor S.J., McLoughlin R.M., Curtis A.M. (2017). The circadian protein BMAL1 in myeloid cells is a negative regulator of allergic asthma. Am. J. Physiol. Lung Cell Mol. Physiol..

[47] Sutton C.E., Finlay C.M., Raverdeau M., JO Early, DeCourcey J., Zaslona Z. (2017). Loss of the molecular clock in myeloid cells exacerbates T cell-mediated CNS autoimmune disease. Nat. Commun..

[48] Hemmers S., Rudensky A.Y. (2015). The cell-intrinsic circadian clock is dispensable for lymphocyte differentiation and function. Cell Rep..

[49] Suzuki K., Hayano Y., Nakai A., Furuta F., Noda M. (2016). Adrenergic control of the adaptive immune response by diurnal lymphocyte recirculation through lymph nodes. J. Exp. Med..

[50] Nakai A., Hayano Y., Furuta F., Noda M., Suzuki K. (2014). Control of lymphocyte egress from lymph nodes through beta2-adrenergic receptors. J. Exp. Med..

[51] He W., Holtkamp S., Hergenhan S.H., Kraus K., de Juan A., Weber J. (2018). Circadian expression of migratory factors establishes lineage-specific signatures that guide the homing of leukocyte subsets to tissues. Immunity.

[52] Shimba A., Cui G., Tani-Ichi S., Ogawa M., Abe S., Okazaki F. (2018). Glucocorticoids drive diurnal oscillations in T cell distribution and responses by inducing interleukin-7 receptor and CXCR4. Immunity.

[53] Vitaterna M.H., King D.P., Chang A.M., Kornhauser J.M., Lowrey P.L., McDonald J.D. (1994). Mutagenesis and mapping of a mouse gene, Clock, essential for circadian behavior. Science.

[54] Nader N., Chrousos G.P., Kino T. (2009). Circadian rhythm transcription factor CLOCK regulates the transcriptional activity of the glucocorticoid receptor by acetylating its hinge region lysine cluster: potential physiological implications. FASEB J..

[55] Spengler M.L., Kuropatwinski K.K., Comas M., Gasparian A.V., Fedtsova N., Gleiberman A.S. (2012). Core circadian protein CLOCK is a positive regulator of NF-kappaB-mediated transcription. Proc. Natl. Acad. Sci. U. S. A..

[56] Bellet M.M., Deriu E., Liu J.Z., Grimaldi B., Blaschitz C., Zeller M. (2013). Circadian clock regulates the host response to Salmonella. Proc. Natl. Acad. Sci. U. S. A..

[57] Wang T., Wang Z., Yang P., Xia L., Zhou M., Wang S. (2016). PER1 prevents excessive innate immune response during endotoxin-induced liver injury through regulation of macrophage recruitment in mice. Cell Death Dis..

[58] Liu J., Malkani G., Shi X., Meyer M., Cunningham-Runddles S., Ma X. (2006). The circadian clock Period 2 gene regulates gamma interferon production of NK cells in host response to lipopolysaccharide-induced endotoxic shock. Infect. Immun..

[59] Arjona A., Sarkar D.K. (2006). The circadian gene mPer2 regulates the daily rhythm of IFN-gamma. J. Interferon Cytokine Res. : Off. J. Int. Soc. Interferon Cytokine Res..

[60] Heipertz E.L., Harper J., Lopez C.A., Fikrig E., Hughes M.E., Walker W.E. (2018). Circadian rhythms influence the severity of sepsis in mice via a TLR2-dependent, leukocyte-intrinsic mechanism. J. Immunol. (Baltimore, Md : 1950).

[61] Nakamura Y., Harama D., Shimokawa N., Hara M., Suzuki R., Tahara Y. (2011). Circadian clock gene Period2 regulates a time-of-day-dependent variation in cutaneous anaphylactic reaction. J. Allergy Clin. Immunol..

[62] Schmutz I., Ripperger J.A., Baeriswyl-Aebischer S., Albrecht U. (2010). The mammalian clock component PERIOD2 coordinates circadian output by interaction with nuclear receptors. Genes Dev..

[63] Hashiramoto A., Yamane T., Tsumiyama K., Yoshida K., Komai K., Yamada H. (2010). Mammalian clock gene Cryptochrome regulates arthritis via proinflammatory cytokine TNF-alpha. J. Immunol. (Baltimore, Md : 1950).

[64] Narasimamurthy R., Hatori M., Nayak S.K., Liu F., Panda S., Verma I.M. (2012). Circadian clock protein cryptochrome regulates the expression of proinflammatory cytokines. Proc. Natl. Acad. Sci. U. S. A..

[65] Cao Q., Zhao X., Bai J., Gery S., Sun H., Lin D.C. (2017). Circadian clock cryptochrome proteins regulate autoimmunity. Proc. Natl. Acad. Sci. U. S. A..

[66] Lam M.T., Cho H., Lesch H.P., Gosselin D., Heinz S., Tanaka-Oishi Y. (2013). Rev-Erbs repress macrophage gene expression by inhibiting enhancer-directed transcription. Nature.

[67] Pourcet B., Zecchin M., Ferri L., Beauchamp J., Sitaula S., Billon C. (2018). Nuclear receptor subfamily 1 group D member 1 regulates circadian activity of NLRP3 inflammasome to reduce the severity of fulminant hepatitis in mice. Gastroenterology.

[68] Wang S., Lin Y., Yuan X., Li F., Guo L., Wu B. (2018). REV-ERBalpha integrates colon clock with experimental colitis through regulation of NF-kappaB/NLRP3 axis. Nat. Commun..

[69] Gagnidze K., Hajdarovic K.H., Moskalenko M., Karatsoreos I.N., McEwen B.S., Bulloch K. (2016). Nuclear receptor REV-ERBalpha mediates circadian sensitivity to mortality in murine vesicular stomatitis virus-induced encephalitis. Proc. Natl. Acad. Sci. U. S. A..

[70] Sato S., Sakurai T., Ogasawara J., Takahashi M., Izawa T., Imaizumi K. (2014). A circadian clock gene, Rev-erbalpha, modulates the inflammatory function of macrophages through the negative regulation of Ccl2 expression. J. Immunol. (Baltimore, Md : 1950).

[71] Pariollaud M., Gibbs J.E., Hopwood T.W., Brown S., Begley N., Vonslow R. (2018). Circadian clock component REV-ERBalpha controls homeostatic regulation of pulmonary inflammation. J. Clin. Investig..

[72] Stapleton C.M., Jaradat M., Dixon D., Kang H.S., Kim S.C., Liao G. (2005). Enhanced susceptibility of staggerer (RORalphasg/sg) mice to lipopolysaccharide-induced lung inflammation. Am. J. Physiol. Lung Cell Mol. Physiol..

[73] Dzhagalov I., Giguere V., He Y.W. (2004). Lymphocyte development and function in the absence of retinoic acid-related orphan receptor alpha. J. Immunol. (Baltimore, Md : 1950).

[74] Delerive P., Monte D., Dubois G., Trottein F., Fruchart-Najib J., Mariani J. (2001). The orphan nuclear receptor ROR alpha is a negative regulator of the inflammatory response. EMBO Rep..

[75] Cavadini G., Petrzilka S., Kohler P., Jud C., Tobler I., Birchler T. (2007). TNF-alpha suppresses the expression of clock genes by interfering with E-box-mediated transcription. Proc. Natl. Acad. Sci. U. S. A..

[76] Okada K., Yano M., Doki Y., Azama T., Iwanaga H., Miki H. (2008). Injection of LPS causes transient suppression of biological clock genes in rats. J. Surg. Res..

[77] Marpegan L., Bekinschtein T.A., Costas M.A., Golombek D.A. (2005). Circadian responses to endotoxin treatment in mice. J. Neuroimmunol..

[78] Marpegan L., Leone M.J., Katz M.E., Sobrero P.M., Bekinstein T.A., Golombek D.A. (2009). Diurnal variation in endotoxin-induced mortality in mice: correlation with proinflammatory factors. Chronobiol. Int..

[79] Beynon A.L., Coogan A.N. (2010). Diurnal, age, and immune regulation of interleukin-1beta and interleukin-1 type 1 receptor in the mouse suprachiasmatic nucleus. Chronobiol. Int..

[80] Kwak Y., Lundkvist G.B., Brask J., Davidson A., Menaker M., Kristensson K. (2008). Interferon-gamma alters electrical activity and clock gene expression in suprachiasmatic nucleus neurons. J. Biol. Rhythm..

[81] Tong X., Buelow K., Guha A., Rausch R., Yin L. (2012). USP2a protein deubiquitinates and stabilizes the circadian protein CRY1 in response to inflammatory signals. J. Biol. Chem..

[82] Koyanagi S., Ohdo S. (2002). Alteration of intrinsic biological rhythms during interferon treatment and its possible mechanism. Mol. Pharmacol..

[83] Wang Y., Pati P., Xu Y., Chen F., Stepp D.W., Huo Y. (2016). Endotoxin disrupts circadian rhythms in macrophages via reactive oxygen species. PLoS One.

[84] Silver A.C. (2017). Pathogen-associated molecular patterns alter molecular clock gene expression in mouse splenocytes. PLoS One.

[85] Yamamura Y., Yano I., Kudo T., Shibata S. (2010). Time-dependent inhibitory effect of lipopolysaccharide injection on Per1 and Per2 gene expression in the mouse heart and liver. Chronobiol. Int..

[86] Shimizu T., Watanabe K., Anayama N., Miyazaki K. (2017). Effect of lipopolysaccharide on circadian clock genes Per2 and Bmal1 in mouse ovary. J. Physiol. Sci..

[87] Haspel J.A., Chettimada S., Shaik R.S., Chu J.H., Raby B.A., Cernadas M. (2014). Circadian rhythm reprogramming during lung inflammation. Nat. Commun..

[88] Kiessling S., Dubeau-Laramee G., Ohm H., Labrecque N., Olivier M., Cermakian N. (2017). The circadian clock in immune cells controls the magnitude of Leishmania parasite infection. Sci. Rep..

[89] Godinho-Silva C., Domingues R.G., Rendas M., Raposo B., Ribeiro H., da Silva J.A. (2019). Light-entrained and brain-tuned circadian circuits regulate ILC3s and gut homeostasis. Nature.

[90] Teng F., Goc J., Zhou L., Chu C., Shah M.A., Eberl G. (2019). A circadian clock is essential for homeostasis of group 3 innate lymphoid cells in the gut. Sci. Immunol..

[91] Nakamura Y., Nakano N., Ishimaru K., Hara M., Ikegami T., Tahara Y. (2014). Circadian regulation of allergic reactions by the mast cell clock in mice. J. Allergy Clin. Immunol..

[92] Wang Q., Robinette M.L., Billon C., Collins P.L., Bando J.K., Fachi J.L. (2019). Circadian rhythm-dependent and circadian rhythm-independent impacts of the molecular clock on type 3 innate lymphoid cells. Sci. Immunol..

[93] Westfall S., Aguilar-Valles A., Mongrain V., Luheshi G.N., Cermakian N. (2013). Time-dependent effects of localized inflammation on peripheral clock gene expression in rats. PLoS One.

